# Dual expression of plastidial GPAT1 and LPAT1 regulates triacylglycerol production and the fatty acid profile in *Phaeodactylum tricornutum*

**DOI:** 10.1186/s13068-018-1317-3

**Published:** 2018-11-22

**Authors:** Xiang Wang, Hong-Po Dong, Wei Wei, Srinivasan Balamurugan, Wei-Dong Yang, Jie-Sheng Liu, Hong-Ye Li

**Affiliations:** 1 Key Laboratory of Eutrophication and Red Tide Prevention of Guangdong Higher Education Institutes, College of Life Science, Jinan University, Guangzhou, 510632 China; 2 School of Ocean and Meteorology, Guangdong Ocean University, Zhanjiang, 524088 China; 3 State Key Laboratory of Estuarine and Coastal Research, East China Normal University, Shanghai, 200062 China

**Keywords:** Diatoms, GPAT1, LPAT1, Biofuel, Triacylglycerol biosynthesis, Prokaryotic TAG pathway

## Abstract

**Background:**

Metabolic engineering has emerged as a potential strategy for improving microalgal lipid content through targeted changes to lipid metabolic networks. However, the intricate nature of lipogenesis has impeded metabolic engineering. Therefore, it is very important to identify the crucial metabolic nodes and develop strategies to exploit multiple genes for transgenesis. In an attempt to unravel the microalgal triacylglycerol (TAG) pathway, we overexpressed two key lipogenic genes, glycerol-3-phosphate acyltransferase (GPAT1) and lysophosphatidic acid acyltransferase (LPAT1), in oleaginous *Phaeodactylum tricornutum* and determined their roles in microalgal lipogenesis.

**Results:**

Engineered *P. tricornutum* strains showed enhanced growth and photosynthetic efficiency compared with that of the wild-type during the growth phase of the cultivation period. However, both the cell types reached stationary phase on day 7. Overexpression of GPAT1 and LPAT1 increased the TAG content by 2.3-fold under nitrogen-replete conditions without compromising cell growth, and they also orchestrated the expression of other key genes involved in TAG synthesis. The transgenic expression of GPAT1 and LPAT1 influenced the expression of malic enzyme and glucose 6-phosphate dehydrogenase, which enhanced the levels of lipogenic NADPH in the transgenic lines. In addition, GPAT1 and LPAT1 preferred C16 over C18 at the sn-2 position of the glycerol backbone.

**Conclusion:**

Overexpression of GPAT1 together with LPAT1 significantly enhanced lipid content without affecting growth and photosynthetic efficiency, and they orchestrated the expression of other key photosynthetic and lipogenic genes. The lipid profile for elevated fatty acid content (C16-CoA) demonstrated the involvement of the prokaryotic TAG pathway in marine diatoms. The results suggested that engineering dual metabolic nodes should be possible in microalgal lipid metabolism. This study also provides the first demonstration of the role of the prokaryotic TAG biosynthetic pathway in lipid overproduction and indicates that the fatty acid profile can be tailored to improve lipid production.

**Electronic supplementary material:**

The online version of this article (10.1186/s13068-018-1317-3) contains supplementary material, which is available to authorized users.

## Background

*Phaeodactylum tricornutum* is a unicellular model pennate diatom that can accumulate lipids particularly when it is subjected to environmental stresses [1–5]. Recently, *P. tricornutum* has emerged as a model candidate for lipid enhancement by metabolic engineering, owing to its high lipid content and the availability of a sequenced genome and genetic tool kit. However, engineered strains accumulate lipids to less than their theoretical maximum [6, 7] and contain a wide range of fatty acid moieties that hinder their commercial exploitation [8, 9]. Furthermore, the triacylglycerol (TAG) biosynthetic pathway is complex and is regulated by various metabolic nodes. Therefore, there is a pressing need to identify the key metabolic nodes and unravel their regulatory mechanisms so that the full potential of microalgal fuel production potential can be realized.

Triacylglycerols in microalgae are primarily stored in lipid droplets (LDs) and serve as energy reservoirs [10]. In microalgae, TAG can be synthesized via the de novo synthetic pathway, the fatty acyl-CoA-dependent Kennedy pathway, and/or the acyl-CoA independent PDAT pathway. The Kennedy pathway comprises three sequential acylations of a glycerol backbone that are catalyzed by glycerol-3-phosphate acyltransferase (GPAT), lysophosphatidic acid acyltransferase (LPAT), and diacylglycerol acyltransferase (DGAT) [11]. The GPAT, LPAT, and DGAT transfer the acyl moiety to the sn-1, sn-2, and sn-3 positions of a glycerol backbone for TAG assembly, respectively. This leads to the generation of lysophosphatidic acid (LPA), phosphatidic acid (PA), diacylglycerol (DAG), and the end product TAG. In higher plants, TAG is assembled in the endoplasmic reticulum (ER) from its precursor DAG and stored in cytosolic LDs. However, in *Chlamydomonas reinhardtii*, Fan et al. [12] found that TAG is produced from DAG in the chloroplast and accumulated in both the chloroplasts and cytosol, thus implying the existence of prokaryotic plastid and eukaryotic ER-localized TAG biosynthetic pathways.

GPAT and LPAT catalyze the initial committed steps. This leads to the formation of key substrates for TAG biosynthesis, and hence, they are considered to be the rate-limiting enzymes [13, 14]. In higher plants, ten GPAT isoforms have been identified, of which two have been reported to be involved in the Kennedy pathway [15–17]. In the model diatom *P. tricornutum*, GPAT overexpression significantly enhances TAG content and alters the fatty acid profile [18]. Our previous study showed that overexpression of LPAT significantly increases TAG content and polyunsaturated fatty acid levels in *P. tricornutum* [19]. Plastidial LPAT has prokaryotic catalytic activity and prefers C16 fatty acids, whereas ER-localized LPAT prefers C18 fatty acids [20–22]. Interestingly, *Chlamydomonas* and related green algae have been shown to possess ER-LPAT, but they exhibit greater activity on C16:0 as plastidial LPATs [23]. Therefore, it is important to understand the acyl-specific catalytic mechanism underlying algal LPAT as well as its pivotal role in producing TAG.

We introduced both *GPAT1* and *LPAT1* genes into *P. tricornutum* cells to produce microalgal strains with high lipid yields. Molecular analyses revealed the expression of the two genes in the transgenic lines. We obtained transgenic strains that showed significantly elevated TAG contents without affecting algal biomass. This study also investigated the potential mechanisms underlying lipid accumulation triggered by GPAT1 and LPAT1 overexpression and elucidated the prokaryotic pathway for TAG assembly in diatoms. A presumptive pathway that transports TAG precursors from the chloroplast to the LDs is proposed for diatoms.

## Materials and methods

### Algal strain and culture conditions

*Phaeodactylum tricornutum* (Strain CCMP-2561) was procured from the Provasoli-Guillard National Center for Marine Algae and Microbiota (East Boothbay, USA). It was maintained in f/2 medium at 20 ± 0.5 °C under a 12 h:12 h light/dark cycle with 200 μmol photons m^−2^ s^−1^ irradiance. Before preparation of the transgenic cells, the algal cells were acclimated in modified f/2 medium without Si. In the nitrogen (N) or phosphorus (P)-depleted experiments, the cells grown under N-replete conditions were harvested by centrifugation (4400×*g* for 10 min), washed with an N or P-free medium, and resuspended in N or P-free medium. In the NADPH inhibition experiment, different concentrations of sesamol (0–2 mM) were added to the medium, and the cells were harvested after 48 h treatment for further analysis. The analyses of the samples taken from the media containing different concentrations of sesamol were performed in triplicate. In addition, cells harvested on day 4 were used for biomass analysis while cells harvested on day 7 were used for the determination of lipid productivity.

### Construction and transformation of the double overexpression system

Total RNA was isolated from algal cells harvested on day 7 using a Plant RNA Kit (Omega, USA) and transcribed into cDNA with a HiScript II Reverse Transcriptase Kit (Vazyme, China) according to the manufacturer’s instructions. The full-length coding regions for GPAT1 (Accession No.: XP_002177014.1) and LPAT1 (Accession No.: XP_002176893.1) were amplified using the primers shown in Additional file 1: Table S1. The *GPAT1* and *LPAT1* gene fragments were purified using a gel extraction kit (Omega, USA) and cloned into the expression vectors pHY18 and pHY21, respectively, through the homologous recombination method, using a CloneExpress II One Step Kit (Vazyme, China). The recombinant plasmids (pHY18-GPAT1 and pHY21-LPAT1) were first linearized and then electroporated into algal cells using Gene Pulser Xcell equipment (Bio-Rad, USA) at a 1:1 ratio (w/w) according to Wang et al. [8]. The transformed cells were grown in f/2 liquid medium without antibiotics for at least 2 days, then selected on f/2 solid medium with chloramphenicol (250 mg L^−1^) and zeocin (100 mg L^−1^). Genomic PCR was performed to verify the integration of the expressions containing the GPAT1 and LPAT1 cassettes in the diatom. The PCR method has been previously described by Kang et al. [24]. Briefly, both WT and transgenic cells were harvested, and their genomic DNA was extracted. Then the genomic DNA was used as the template for the PCR. Taq PCR StarMix (GenStar, China) was also used to run the PCR. The antibiotic gene *Shble* and *CAT* in the expression cassette were amplified using primers Shble-f, Shble-r, CAT-f, and CAT-r, whereas the 18S rDNA gene was detected using primers 18s-f and 18s-r (Additional file 1: Table S1). The expected PCR product lengths of *Shble*, *CAT*, and the 18s rDNA were 349 bp, 500 bp, and 498 bp, respectively.

### Quantitative real-time PCR, western blotting, and enzymatic activity assays

Total RNA was extracted for qRT-PCR using a Plant RNA Kit (Omega, USA) and its concentration was measured using a NanoDrop 2000 spectrophotometer (Thermo Scientific, USA). The cDNA was synthesized using HiScript II Q RT SuperMix for qPCR (Vazyme, China). The qRT-PCR reactions were performed in eight-strip real-time PCR tubes containing 20 μL AceQ qPCR SYBR Green Master Mix (Vazyme, China). The relative transcript abundance was calculated by the 2^−ΔΔCt^ method after the expression had been normalized to that of the endogenous housekeeping gene *β*-*actin*. Three biological replicates were analyzed. The primers for the qRT-PCR are listed in Additional file 1: Table S1.

Western blot analysis was used to examine the expression of the target proteins. Total protein was extracted from algal cells with RIPA lysis buffer (Beyotime, China) containing phenylmethanesulfonyl fluoride (PMSF, Beyotime, China). The total protein concentration was determined with a BCA protein quantification kit (Beyotime, China). After quantification, the proteins were first separated by sodium dodecyl sulfate-polyacrylamide gel electrophoresis and then electro-transferred to a polyvinylidene difluoride membrane pre-activated by methanol. The membrane was incubated with primary anti c-Myc antibody (1:3000, Abcam, UK) or anti-flag antibody (1:2000, Sigma, USA) overnight at 4 °C after it had been blocked with skimmed milk for 1 h at 4 °C. This was followed by incubation with HRP-conjugated goat anti-rabbit secondary antibody (1:5000, CST, USA) for 2 h at 4 °C. Then, the membrane was washed three times in pre-cooled PBST (Sangon, China) and developed with a chemiluminescence system (Millipore, USA). Endogenous β-actin was used as an internal control.

The activity levels of GPAT1, LPAT1 and malic enzyme (ME) were measured using a plant GPAT activity spectrophotometry assay kit (Bangyi, China), a plant LPAT activity spectrophotometry assay kit (Bangyi, China), and a ME colorimetric quantitative detection kit (Ke Ming Co., China), respectively, according to the manufacturer’s instructions.

### Measurement of photosynthetic parameters

The effective photochemical efficiency of photosystem II (Fv/Fm) and the electron transport rate (ETR) were measured using a PhytoPAM Phytoplankton Analyzer (Walz, Germany). Non-photochemical quenching (NPQ) was calculated using the formula: NPQ = (Fm − Fm′)/Fm′ [25]. Chlorophyll *a* and *c* were quantified using an Agilent 1200 HPLC system (Agilent Technologies, USA) with a Symmetry C8 column as described previously [26]. Cell density was calculated daily through the direct count method, with a microscope and a bright-line Neubauer hemocytometer. Specific growth rate (*μ*) of culture in log phase was calculated following the equation reported by Nur et al. [27].

### Lipid, protein, and carbohydrate analysis

The relative neutral lipid (NL) content was determined with the Nile-red staining method as described previously with minor modification [28]. Briefly, 50 μL Nile red solution (0.1 mg mL^−1^ in acetone) was added to 5 mL algal culture and incubated for 20 min in the dark at 37 °C. Then the mixture was transferred to a 96-well plate, and the relative fluorescence intensity was measured with a microplate reader (Bio-Tek, USA) at an excitation wavelength of 480 nm and an emission wavelength of 592 nm. Total lipids (TLs) were determined gravimetrically according to a previously described method [8]. Approximately, 50 mg lyophilized algal cells were ground to a powder in liquid nitrogen and transferred to 10 mL tubes. Then 3.8 mL mixed solvent (methanol/chloroform/water, 2:1:0.8, v/v/v) was added, and the mixture was sonicated for 15 min at 200 W. Then 2 mL of chloroform/water (1:1, v/v) was added, and the solution was mildly vortexed. The suspension was separated into two layers by centrifugation at 2000 rpm for 5 min. The upper phase was discarded, and the lower phase was collected in a pre-weighed tube. The extract was dried under a stream of N_2_ and weighed.

The TAG was determined by extracting the total lipids from the algal cells as described as above and dissolving them in chloroform. The lipids were separated by thin layer chromatography on a silica plate, using the developing solvent hexane/diethyl ether/acetic acid (85:15:1, v/v/v). The separated TAG was visualized by iodine vapor, scraped off the plate, and dissolved in chloroform. The solution was centrifuged at 12,000×*g* for 10 min, and the supernatant was collected, dried by N_2_ steam flow, and TAG was gravimetrically determined. Fatty acid composition was determined as fatty acid methyl esters with a gas chromatography–mass spectrophotometer equipped with an NIST 147 spectrum library, according to the method described by Balamurugan et al. [19]. The peak areas were normalized to the internal standard methyl nonadecylate (Aladdin, China).

The total carbohydrate content was obtained following the phenol–sulfuric acid method [29]. Briefly, cell pellets were resuspended in 1 mL ddH_2_O. Then 1 mL 5% phenol solution was added to the mix, followed by 5 mL sulfuric acid (95–98%, v/v). The solution was incubated in a water bath at 25 °C for 10 min, and the absorbance of the solution was read at a wavelength of 483 nm. Based on the curve of glucose standard, carbohydrate content per liter of culture was obtained. This value was divided by cell density to get carbohydrate content per cell. Total soluble protein was extracted with the method described above and determined using a BCA protein quantification kit (Beyotime, China). According to the standard curve of bovine serum albumin, total soluble protein per liter of culture was calculated.

### Confocal microscopy and subcellular localization analysis

One milliliter of culture was stained with 10 μL Nile red solution (0.1 mg mL^−1^ in acetone) and incubated at 37 °C for 30 min in the dark. The stained cells were observed under an LSM880 laser-scanning confocal microscope (Zeiss, Germany) with an excitation wavelength of 514 nm, an emission wavelength of 596 nm, and a detection wavelength range of 539–652 nm.

The subcellular localization of GPAT1 and LPAT1 was determined by fusing EGFP with the 3′-terminus of the target gene in pPhAP1-EGFP following the previous method [30]. The recombinant plasmids containing the target genes were electroporated into algal cells. The cells were observed under an LSM880 confocal laser scanning microscope (Zeiss, Germany), and the EGFP fluorescence was observed at an excitation wavelength of 488 nm and an emission wavelength range of 510–555 nm. Chlorophyll auto-fluorescence was detected at an excitation wavelength of 488 nm and an emission wavelength range of 625–720 nm.

### Statistical analysis

All experiments were carried out in triplicate, and the data are expressed as mean ± SD. All statistical tests were performed using the SPSS statistical package 19.0. Paired *t*-tests were used to compare two groups. The results were considered to be significantly different at *p* < 0.05 (*) or *p* < 0.01 (**).

## Results and discussion

### Sequence analysis and construction of the expression system

The sequence structure of GPAT1 and LPAT1 predicted using SMART (http://smart.embl-heidelberg.de/) [31] and ChloroP (http://www.cbs.dtu.dk/services/ChloroP/) [32] showed that GPAT1 contained a chloroplastic signal peptide, a LPLAT superfamily and two transmembrane regions, whereas LPAT1 had a plastidial signal peptide, a transmembrane region, and a PlsC domain (Additional file 1: Figure S1), thus suggesting that they are both membrane proteins localized to the chloroplast. However, the subcellular localization of GPAT1 and LPAT1 predicted using HECTAR [33] revealed that LPAT1 localized to the chloroplasts, whereas GPAT1 localized to the mitochondria (Additional file 1: Table S2). The results indicated that bioinformatic prediction is inaccurate. To further validate the localization of GPAT1 and LPAT1, we used pPhAP1 with EGFP to express the fusion protein for GPAT1 or LPAT1 (Additional file 1: Figure S2B and D). The confocal microscopy images showed that both GPAT1 and LPAT1 localized to the chloroplasts (Additional file 1: Figure S3), thus verifying the previous immunoelectron microscopy results [18, 19].

The full-length coding sequences for GPAT1 and LPAT1 were PCR amplified and inserted into the vectors pHY-18 and pHY-21, respectively, for co-overexpression (Additional file 1: Figure S2A and C).

### Verification of transgenic strains using molecular approaches

**Fig. 1:**
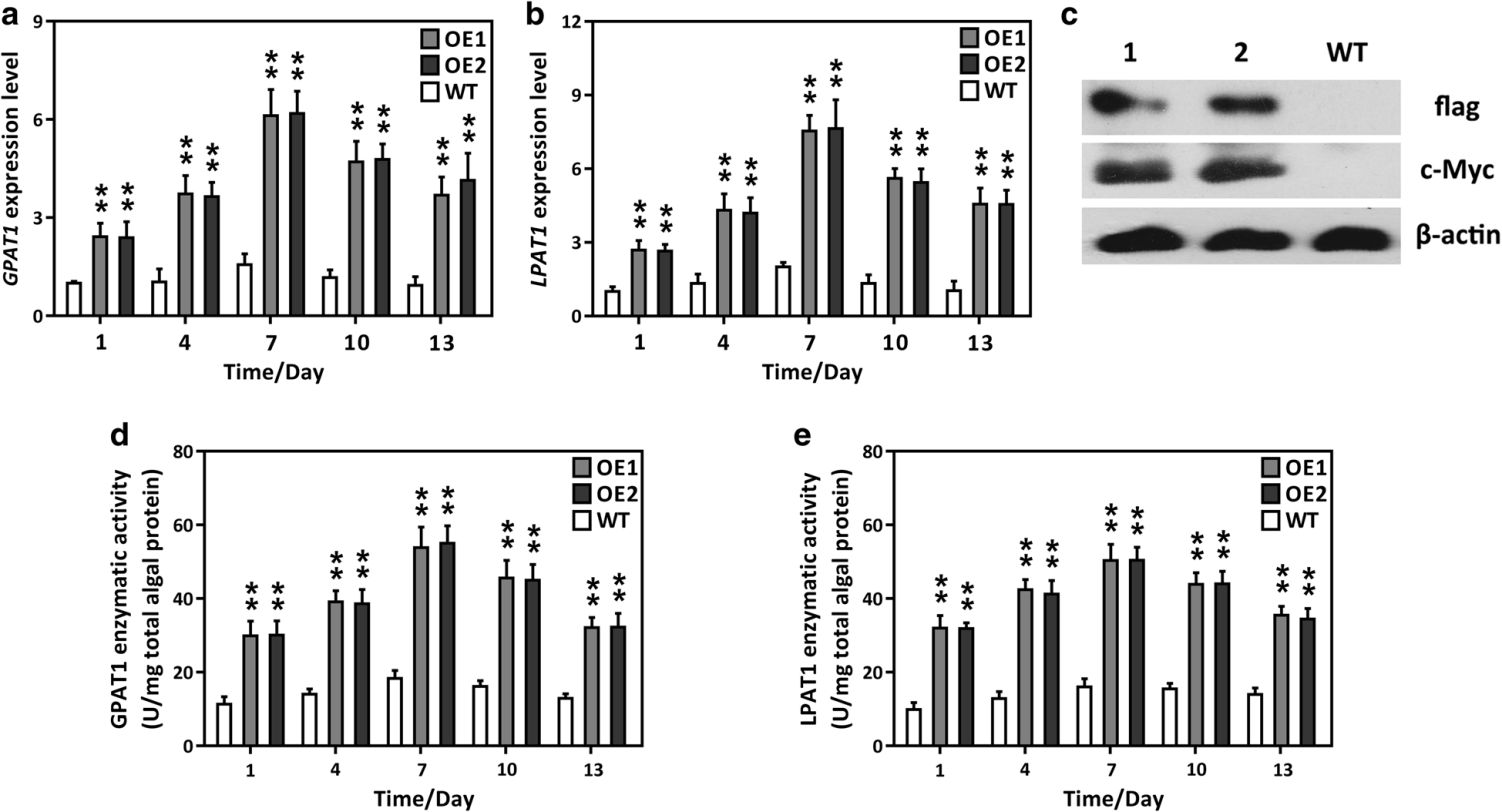
Molecular characteristics of GPAT1 and LPAT1 co-overexpressed lines (OE1 and OE2) and wild-type (WT). **a** Relative expression abundance of *GPAT1* during the whole growth period (13 days), *β-actin* was served as an internal reference gene; **b** relative expression abundance of *LPAT1* during the whole growth period, *β-actin* was served as an internal reference gene; **c** Western blot analysis for detection of c-Myc and flag. Cells were collected on day 7. Lane 1 and 2 represent OE1 and OE2 lines, respectively. β-actin was served as a housekeeping protein; **d** changes of GPAT1 enzymatic activity during the whole growth phase; **e** changes of LPAT1 enzymatic activity in microalgae during the whole growth phase. A significant difference between WT and OE lines is indicated at the *p* < 0.05 (*) or *p* < 0.01 (**) level. Each value represents the mean ± SD (*n* = 3)

Putative transformants were screened using solid medium supplemented with chloramphenicol and zeocin. More than ten independent microalgal colonies were selected and inoculated into a liquid medium with chloramphenicol and zeocin for further screening. Then the cells were transferred to fresh medium without antibiotics for further experimental analysis. Genomic PCR was performed to detect the integration of the expression cassette in the transgenics. As expected, two amplicons (349 bp and 500 bp) were amplified in the two overexpression (OE) lines, OE1 and OE2, whereas no such bands were detected in the wild-type (WT) and negative control (B) (Additional file 1: Figure S4). This result demonstrated that the two expression cassettes harboring GPAT1 and LPAT1 were successfully introduced into *P. tricornutum* cells. Subsequently, qRT-PCR was performed to examine the *GPAT1* and *LPAT1* transcript levels in *P. tricornutum*. Figure 1a, b shows that the relative transcript levels of *GPAT1* and *LPAT1* were significantly higher in the OE lines than in the WT cultures over the 13 day culture period. They reached a maximum at day 7 and were upregulated by 5.11–6.64-fold in the transgenic lines compared with the WT. Interestingly, the expression of both the GPAT1 and LPAT1 transcripts gradually decreased after day 7, possibly because of nutrient depletion. In the two vectors, a c-Myc tag and a 2 × flag tag were ligated to the 3′-terminus of GPAT1 and LPAT1, respectively, so that their protein expression could be detected. Western blot analysis showed that two specific cross-reactive protein bands, which corresponded to the expected protein molecular weights, were present in the OE lines, whereas no such protein bands were detected in the WT cultures (Fig. 1c). These results indicated that the introduced GPAT1 and LPAT1 were successfully expressed in the transgenic cells. In addition, the enzymatic activities of GPAT1 and LPAT1 were assayed. The activities of the two enzymes were considerably higher in the OE lines than the WT, and this increase in activity occurred over the entire growth phase and reached a maximum on day 7 (Fig. 1d, e). These results confirmed that GPAT1 and LPAT1 were functional in the OE lines.

### Effect of GPAT1 and LPAT1 overexpression on photosynthesis

**Fig. 2:**
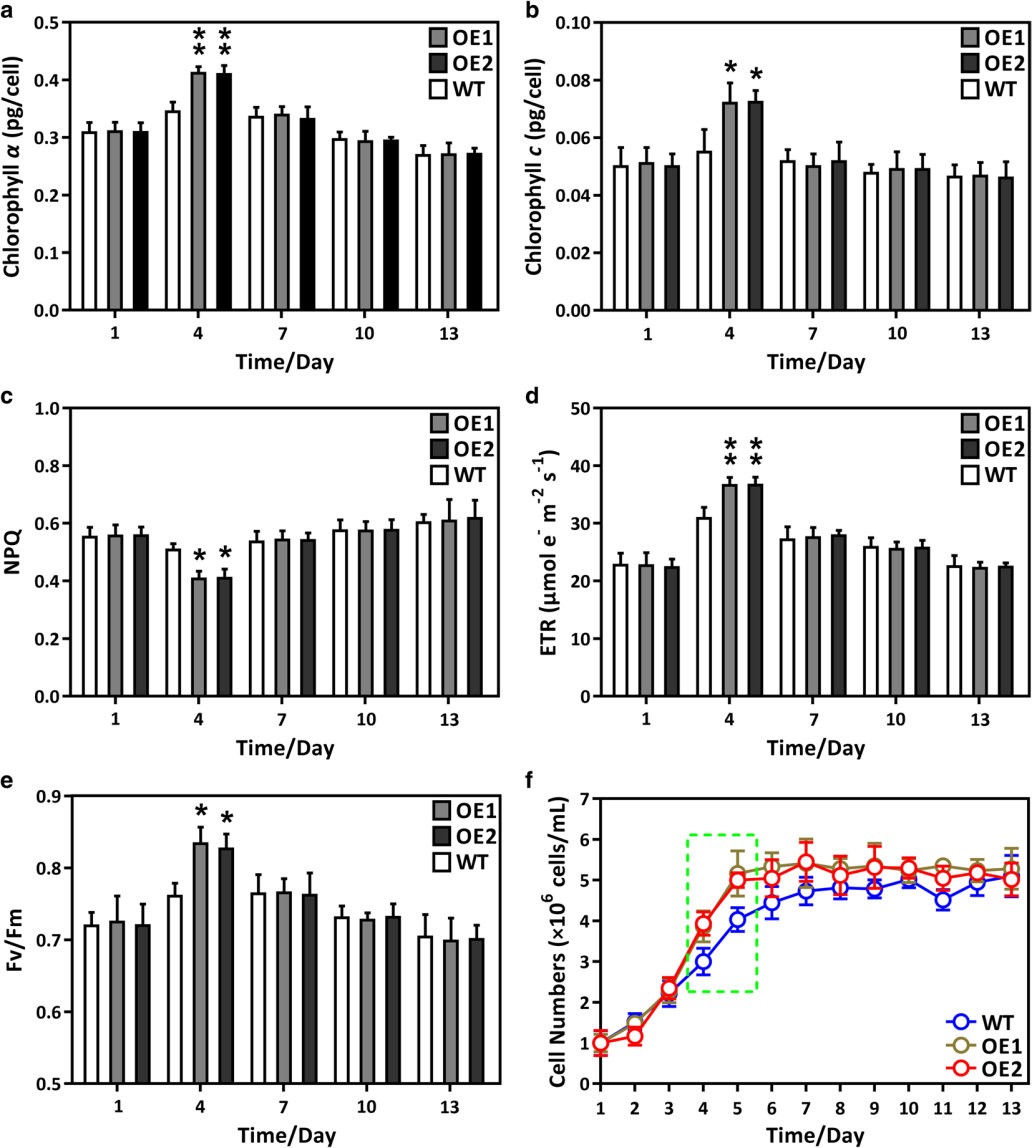
Physiological characterization of GPAT1 and LPAT1 dual overexpression lines (OE1 and OE2) and wild-type (WT) during the whole growth period (13 days). **a** Chlorophyll *α* content; **b** chlorophyll *c* content; **c** non-photochemical quenching (NPQ); **d** electron transport rate (ETR); **e** effective photochemical efficiency of photosystem II (Fv/Fm); **f** growth curve. A significant difference between WT and OE lines is indicated at the *p* < 0.05 (*) or *p* < 0.01 (**) level. Each value represents the mean ± SD (*n* = 3)

Assessment of cellular physiological characteristics is crucial if *P. tricornutum* is to be commercially exploited. In this study, we measured a number of important photosynthetic parameters. Figure 2 shows that chlorophyll *a* or *c* content, the ETR, and Fv/Fm were significantly higher in the OE lines at day 4 than the WT cultures, whereas NPQ was lower. These results indicated that photosynthetic efficiency was elevated in the OE lines, in accordance with the enhanced growth rate of the OE lines during the mid-log phase (green dotted frame in Fig. 2f, Table 1). We also determined the transcript abundance of two key genes, phosphatidic acid phosphohydrolase (*PAP*) and monogalactosyldiacylglycerol synthase (*MGD*), which directly participate in diacylglycerol (DAG) and monogalactosyldiacylglycerol (MGDG) biosynthesis. Interestingly, the *PAP* and *MGD* transcription levels were significantly higher in the OE lines than the WT cultures after day 4 (Fig. 5i, p), which suggested that the DAG and MGDG content in the OE lines had also increased. In algae, DAG is the precursor of thylakoid membrane lipids [34, 35], which are crucial for high-efficiency photosynthesis [36–39]. It is likely that the overproduction of DAG and membrane lipids, triggered by GPAT1 and LPAT1 coordinated overexpression, might have led to the higher photosynthetic efficiency in OE cells. It has been reported that in *A. thaliana*, increased levels of thylakoid membrane lipids elevate photosynthetic efficiency [40]. In addition, to further support the notion, we determined the content of glycolipids and found that cellular glycolipid content was significantly higher in the OE lines at day 4 than the WT cultures (Additional file 1: Figure S5). Glycolipids are main components in thylakoid membrane lipids and have been shown to play a crucial role in maintaining photosynthetic performance [41–43]. Thus, the elevated photosynthetic efficiency in the OE lines may be related to the increase in glycolipids.

**Table 1:** Specific growth rate, biomass and lipid productivity of overexpression lines (OE-1 and OE-2) and wild-type (WT)

Content	Strains		
	WT	OE-1	OE-2
Specific growth rate (μ)^a^	0.542 ± 0.069	0.772 ± 0.093**	0.785 ± 0.078**
Biomass (DCW, g L^−1^)	1.284 ± 0.132	1.671 ± 0.151**	1.664 ± 0.177**
Total lipid productivity (mg day^−1^ 10^9^ cells^−1^)	1.023 ± 0.071	2.737 ± 0.195**	2.779 ± 0.209**
TAG productivity (mg day^−1^ 10^9^ cells^−1^)	0.689 ± 0.053	1.953 ± 0.142**	2.037 ± 0.139**

^a^Determined in the log phase. Specific growth rate and biomass were measured on day 4 while total lipid productivity and TAG productivity were determined on day 7. Each value represents the mean ± SD (*n* = 3). A significant difference between WT and OE lines is indicated at the *p* < 0.05 (*) or *p* < 0.01 (**) level

### Co-overexpression altered the contents of cellular components

**Fig. 3:**
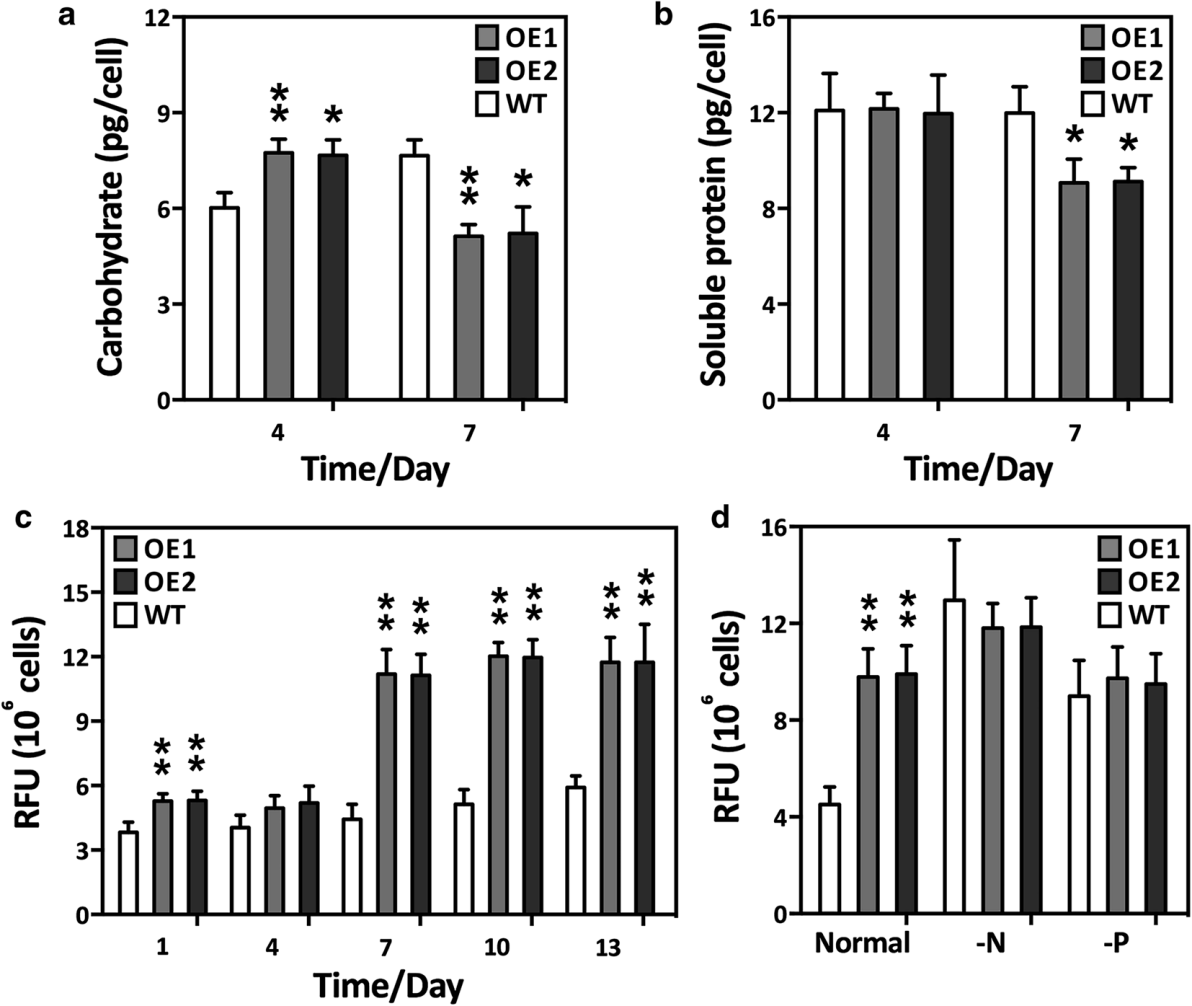
Primary metabolites and relative neutral lipid content of overexpression lines (OE1 and OE2) and wild-type (WT). **a** Total carbohydrate content of cells harvested on day 4 and 7; **b** soluble protein content of cells collected on day 4 and 7. **c** relative neutral lipid content of GPAT1 and LPAT1 overexpression lines (OE) using Nile-red staining method under normal nutrient condition during the whole growth period (13 days); **d** relative neutral lipid content in cells under normal condition (Normal) and cells subjected to 48 h of N (-N) or P (-P) deprivation. Cells were harvested on day 7 and subsequently placed into nutrient-replete and depleted media for 48 h of culture, respectively. A significant difference between WT and OE lines is indicated at the *p* < 0.05 (*) or *p* < 0.01 (**) level. Each value represents the mean ± SD (*n* = 3)

**Fig. 4:**
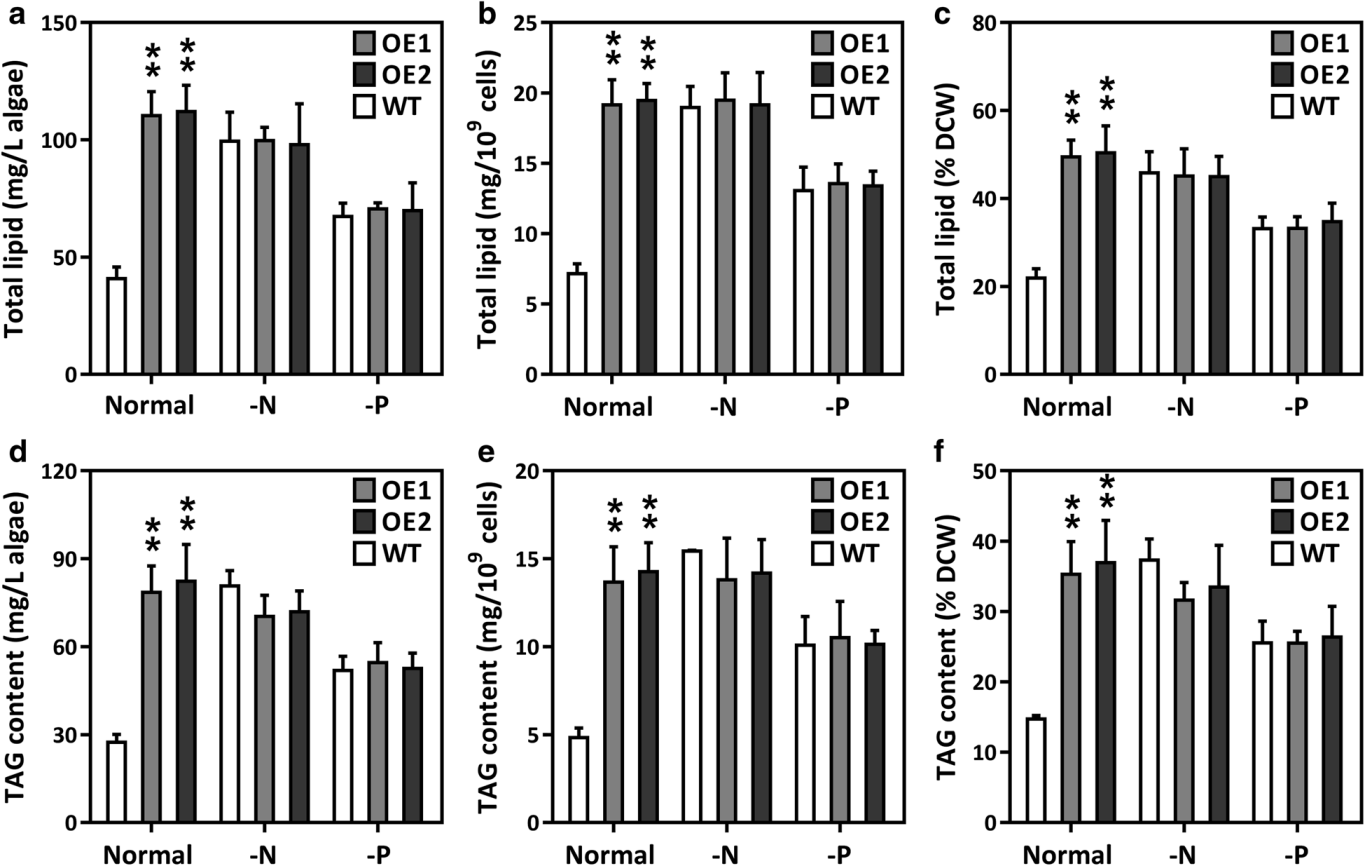
Total lipid content of overexpression lines (OE1 and OE2) and wild-type (WT) under normal condition (Normal) and cells subjected to 48 h of N (-N) or P (-P) deprivation. Cells were harvested on day 7 and subsequently placed into nutrient-replete and depleted media for 48 h of culture, respectively. **a** Total lipid content per liter of algal culture; **b** total lipid content per 10^9^ algal cells; **c** total lipid content per mg dry weight; **d**, triacylglycerol (TAG) content per liter of algal culture; **e** TAG content per 10^9^ algal cells; **f** TAG content per mg dry weight. A significant difference between WT and OE lines is indicated at the *p* < 0.05 (*) or *p* < 0.01 (**) level. Each value represents the mean ± SD (*n* = 3)

The total carbohydrate and soluble protein contents of the OE lines and WT cultures were analyzed at days 4 and 7. Figure 3a shows that the total carbohydrate content was significantly higher in the OE lines than the WT cultures on day 4, but decreased by day 7. There was no significant difference in soluble protein between the WT cultures and the OE lines on day 4, but soluble protein in the OE lines suddenly decreased on day 7 (Fig. 3b). We hypothesize that during the mid-log cultivation phase, the elevated photosynthesis in the OE lines might have required an increased CO_2_ supply and, therefore, more of the carbon flux was directed towards carbohydrate synthesis. However, in the late-log phase, carbon precursors and energy sources might have been redirected to lipogenesis in the OE lines. In cyanobacteria, sucrose permease (encoded by *CscB*), a proton symporter, is involved in the transportation of sucrose into the cell across the cell membrane through proton symport [44]. *CscB* overexpression led to significant increases in biomass, photosystem II activity, and chlorophyll content, thus indicating that elevated photosynthesis redirects carbon flux to carbohydrate biosynthesis in cyanobacteria [44]. The results also confirmed that the biomass of the OE lines also increased, in accordance with the elevated photosynthesis levels (Table 1). Cellular lipids were also analyzed. The relative NL, TL, and TAG content was considerably higher in the OE lines after day 7 than in the WT culture (Figs. 3c, d and 4). This result suggests that overexpression of GPAT1 and LPAT1 effectively promoted the accumulation of cellular lipids, especially TAG. The dual overexpression lines had a higher total lipid content (50.4% of dry biomass) than the single over-expression GPAT1 or LPAT1 cell lines used in previous studies (lipid levels 42.6% or 42.9% of dry biomass, respectively) [18, 19]. Laser scanning confocal microscopic analysis of Nile-red stained cells indicated that the volume of the LDs was significantly higher in the OE lines than the WT cultures (Additional file 1: Figure S6). This result was similar to an observation by Talebi et al. [45] in which the carbon flux from starch was found to be channeled to fatty acid synthesis when AccD and ME were overexpressed in *Dunaliella salina*. There were no significant changes in NL, TL, and TAG under the -N or -P conditions (Fig. 4). Therefore, the double overexpression OE lines might potentially reach their maximum lipid accumulation under normal nutrient conditions. The results from this study are in accordance with our previous observation that cells cease to accumulate lipids after the lipid content reaches a maximum [46, 47]. Additionally, the *P. tricornutum* lipid productivity was calculated to assess whether *P. tricornutum* could potentially be used as a feedstock for biofuels. Table 1 shows that total lipid productivity was 1.023 ± 0.071 mg day^−1^ 10^9^ cells^−1^ for WT, whereas it reached 2.737 ± 0.195 mg day^−1^ 10^9^ cells^−1^ and 2.779 ± 0.209 mg day^−1^ 10^9^ cells^−1^, respectively, for the two OE lines. There was also a significant TAG productivity increase in the OE lines. Altogether, the data demonstrated that double overexpression strains may potentially increase lipid production without altering cellular biomass.

### Effect of collective GPAT1 and LPAT1 overexpression on key genes involved in photosynthesis and TAG synthesis

**Fig. 5:**
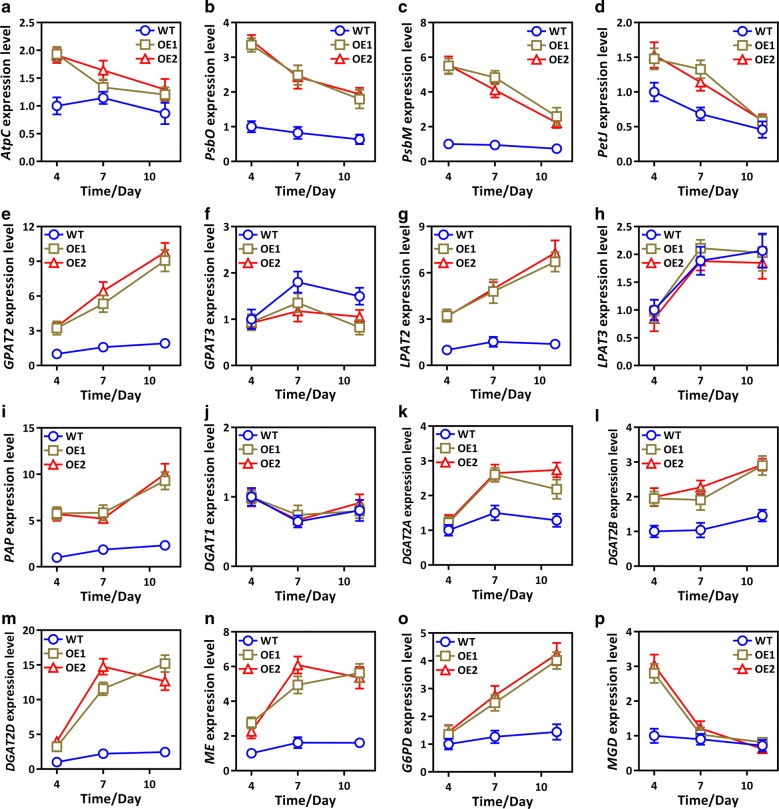
Relative expression levels of genes related to photosynthesis, lipid and NADPH biosynthesis in overexpression lines (OE1 and OE2) and wild-type (WT). **a**–**d** Photosynthetic related genes including ATPase gamma subunit (*AtpC*), oxygen-evolving enhancer protein (*PsbO*), photosystem II subunit (*PsbM*) and cytochrome c6 (*PetJ*); **e**–**m** TAG biosynthetic related genes including glycerol-3-phosphate acyltransferase 2 (*GPAT2*), glycerol-3-phosphate acyltransferase 3 (*GPAT3*), lysophosphatidyl acyltransferase 2 (*LPAT2*), lysophosphatidyl acyltransferase 3 (*LPAT3*), phosphatidic acid phosphatase (*PAP*), diacylglycerol acyltransferase 1 (*DGAT1*), diacylglycerol acyltransferase 2A (*DGAT2A*), diacylglycerol acyltransferase 2B (*DGAT2B*) and diacylglycerol acyltransferase 2D (*DGAT2D*); **n**, **o** NADPH biosynthetic related genes including malic enzyme (*ME*) and glucose-6-phosphate dehydrogenase (*G6PD*); **p** *MGD*, a monogalactosyldiacylglycerol synthase gene. Each value represents the mean ± SD (*n* = 3). The gene information and primers are shown in Additional file 1: Table S1. Each value represents the mean ± SD (*n* = 3)

Co-overexpression has a crucial role in controlling photosynthetic efficiency and lipid content. Therefore, we attempted to elucidate the effect of double overexpression on the expression of other key genes involved in photosynthesis and lipogenesis. Figure 5a–d shows that the photosynthetic related genes *AtpC*, *PsbO*, *PsbM*, and *PetJ* were significantly higher in OE lines on day 4 than in the WT cultures, but after mid-log phase, their expression declined. Furthermore, genes involved in TAG biosynthesis, including *GPAT2*, *LPAT2*, *PAP*, *DGAT2A*, *DGAT2B*, and *DGAT2D*, were detected during the stationary phase (Fig. 5), and their transcript abundance was significantly higher in the OE lines than the WT cultures. The data further suggested that photosynthesis was higher during the growth phase and that the resultant photosynthetic carbon was potentially used for lipogenesis during the stationary phase. Interestingly, the expression of *GPAT3* and *LPAT3*, which were predicted to be localized to the ER, was not significantly different between the WT cultures and OE lines, which suggested that the ER-localized TAG biosynthetic pathway was not affected by the double overexpression of these genes. The results also indicated that the plastidial TAG biosynthetic pathway has a promising role in lipid accumulation. Malic enzyme and glucose-6-phosphate dehydrogenase play crucial roles in providing lipogenic NADPH to microalgae [48–50]. Therefore, this study examined whether double overexpression might also control the expression of NADPH-associated genes in OE lines. Interestingly, the transcript expression of *G6PD* and particularly *ME* was higher in the OE lines than the WT cultures after day 4 (Fig. 5n, o). However, the mechanism underlying such regulation remains unclear. Sesamol, a potent inhibitor of ME activity and NADPH supply, was used to determine the relationships among NADPH, lipid content, and ME expression in OE lines [51, 52]. The results showed that 1.5 mM sesamol significantly reduced the increase in ME expression levels, ME enzyme activity and NADPH content in the OE lines, which in turn led to a decrease in lipid accumulation in the OE lines (Additional file 1: Figure S7). These analyses revealed that double overexpression elevated NADPH content and showed that ME has a significant role in providing lipogenic NADPH.

### TAG synthesis mediated by GPAT1 and LPAT1

**Fig. 6:**
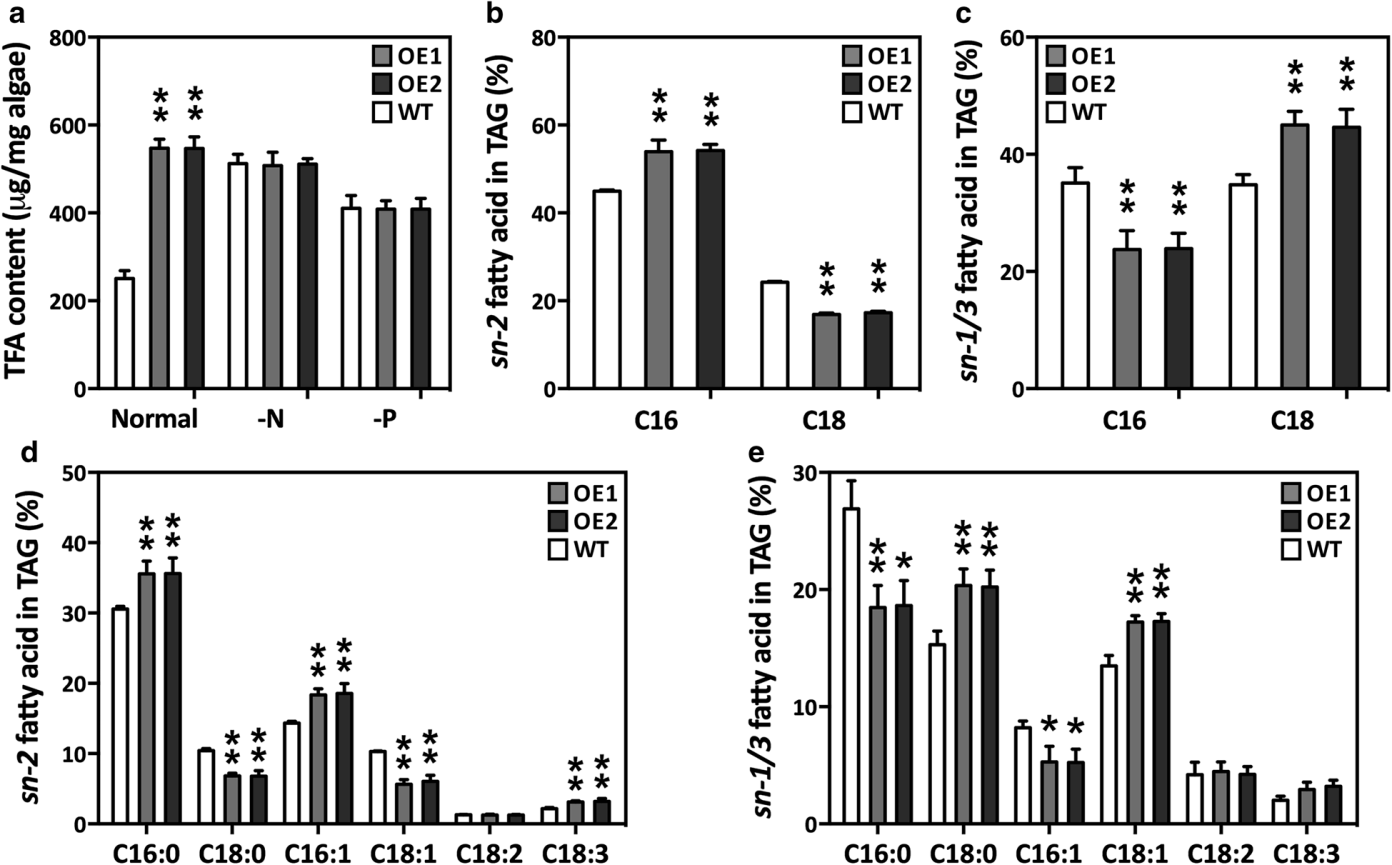
Fatty acid content of overexpression lines (OE1 and OE2) and wild-type (WT). **a** Total fatty acid (TFA) content in cells under normal condition (normal) and cells subjected to 48 h of N (-N) or P (-P) deprivation. Cells were harvested on day 7 and subsequently placed into nutrient-replete and depleted media for 48 h of culture, respectively; **b**, **d** relative abundance of main fatty acids in *sn*-*2* position of TAG in cells harvested on day 7; **c** relative abundance of total C16 and C18 fatty acids in *sn*-*1*/*3* position of TAG in cells harvested on day 7; **e** relative abundance of main fatty acids in *sn*-*1*/*3* position of TAG in cells harvested on day 7. C16 represents the sum of C16:0 and C16:1 while C18 represents the sum of C18:0, C18:1, C18:2 and C18:3. Each value represents the mean ± SD (*n* = 3). A significant difference between WT and OE lines is indicated at the *p* < 0.05 (*) or *p* < 0.01 (**) level. Each value represents the mean ± SD (*n* = 3)

In microalgae, the fatty acyls for TAG assembly can be both de novo synthesized and recycled from membrane polar lipids. Our results showed that there was a significant increase in TAG content when the fatty acid content increased, which suggested that de novo synthesized fatty acids have an important role in TAG assembly (Fig. 6a). This study and previous studies [18, 19] showed that GPAT1 and LPAT1 are localized in the diatom chloroplasts. However, the confocal microscopy images revealed that LDs were present in the cytoplasm and that most of them surrounded the chloroplast (Additional file 1: Figure S6). Furthermore, it is unclear how overexpression of GPAT1 and LPAT1 led to enlarged LDs in the cytoplasm. Fan et al. [12] suggested that *C. reinhardtii* can utilize DAG from chloroplasts to synthesize TAG, and that the TAG formed in this way is deposited in both the chloroplasts and the cytoplasm LDs. However, it has been recently shown that these chloroplast LDs in *C. reinhardtii* are actually LDs implanted within the chloroplast invaginations, which are part of the outer membrane of the chloroplast [53]. Therefore, we inferred that, like *C. reinhardtii*, diatoms may use a pathway that transports TAG precursors, such as DAG, from the chloroplasts to the LDs through a close association with the outer membrane of the chloroplast.

**Fig. 7:**
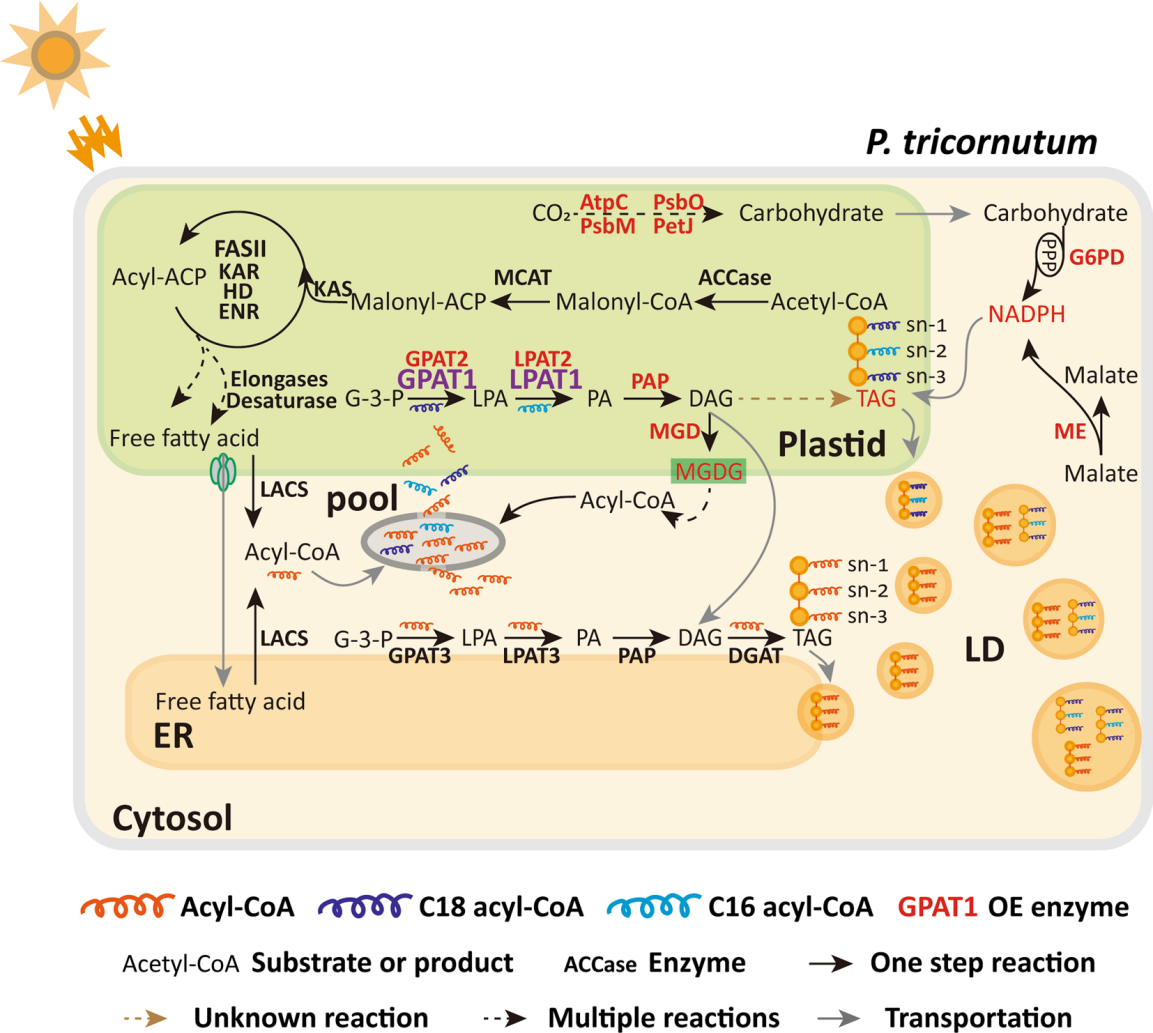
Proposed pathway for TAG assembly in GPAT1 and LPAT1 overexpression lines of *P. tricornutum*. Regular font represents substrate or product; bold font represents enzyme; red bold font represents upregulated enzyme; purple bold font represents overexpressed enzyme. *ACCase* acetyl CoA carboxylase, *MCAT* malonyl-CoA:ACP transacylase, *KAS* 3-ketoacyl-ACP synthase, *KAR* 3-ketoacyl-ACP reductase, *HD* 3-hydroxyacyl-ACP dehydratase, *ENR* enoyl-ACP reductase, *FAT* fatty acyl-ACP thioesterase, *LACS* long-chain acyl-CoA synthetase, *MGDG* monogalactosyldiacylglycerol, *MGD* MGDG synthases, *GPAT* glycerol-3-phosphate acyltransferase, *LPAT* lysophosphatidate acyltransferase, *PAP* phosphatidic acid phosphatase, *DGAT* diacylglycerol acyltransferase, *AtpC* ATPase gamma subunit, *PsbO* oxygen-evolving enhancer protein, *PsbM* photosystem II subunit, *PetJ* cytochrome c6, *ME* malic enzyme, *G6PD* glucose-6-phosphate dehydrogenase, *G-3-P* glycerol-3-phosphate, *LPA* lysophosphatidic acid, *PA* phosphatidic acid, *DAG* diacylglycerol, *TAG* triacylglycerol, *LD* lipid droplet

In vascular plants, glycerolipids are assembled by prokaryotic or eukaryotic pathways. In the prokaryotic pathway, a 16-carbon acyl group is incorporated into the sn-2 position of glycerolipids in plastids by the substrate-specific activity of plastidial LPAT, whereas in the eukaryotic pathway, an 18-carbon acyl group is added to the sn-2 position of glycerolipids in the ER [54]. The results from this study demonstrated that C16:0 dominated the TAG sn-2 position (Fig. 6b, d), which suggested that the sn-2 fatty acids in diatom TAGs are mainly assembled by the prokaryotic pathway. Similar observations have been found in green algae [12, 55–57]. In addition, the relative content of C16:0 and C16:1 at the TAG sn-2 position was significantly higher in the OE lines than the WT cultures (Fig. 6d), which suggested that LPAT1 may contribute more to prokaryotic TAG biosynthesis in diatoms. The phylogenetic analyses revealed that LPAT1 in *P. tricornutum*, CrLPAAT1 in *C. reinhardtii*, and LPAT1 in *A. thaliana* belonged to the same clade and were divergent from the ER-located CrLPAAT2 in *C. reinhardtii* (Additional file 1: Figure S8). In *C. reinhardtii*, the plastid-located CrLPAAT1 prefers 16:0-CoA to 18:1-CoA as an acyl donor [22]. These results suggest that the plastidial LPAT1 in *P. tricornutum* may have a similar function to the CrLPAAT1 in *C. reinhardtii*. C18:0 and C18:1 were significantly higher at the TAG sn-1/sn-3 position in the OE lines than the WT cultures, whereas C16:0 and C16:1 were significantly lower (Fig. 6c, e). The TAG positional analysis demonstrated that double overexpression resulted in differential changes in the fatty acid profiles of the TAG sn-2 and sn-1/sn-3 positions. In *C. reinhardtii*, the C18 fatty acids were more abundant than the C16 acyl chains at the sn-1/sn-3 position in TAG, which suggested that the availability of fatty acids or substrate specificity of acyltransferases may play an important role in cells [12]. The results from this study indicated that the C18 fatty acid percentage content was higher than the C16 percentage content in the OE lines, but they were very similar in the WT cultures. It is probable that more C18 fatty acids were available or preferred in the double overexpression system. Based on the aforementioned discussion, a schematic pathway for TAG assembly and LD formation in GPAT1 and LPAT1 overexpression cells is proposed (Fig. 7).

## Conclusion

In this study, we report the functional importance of the TAG biosynthetic machinery in the overproduction of engineered fatty acids after the double overexpression of GPAT1 and LPAT1 in oleaginous *P. tricornutum*. The TAG content and maximum lipid content was considerably elevated in the OE lines, but cellular biomass did not decrease. GPAT1 and LPAT1 localized to plastids. Their increased expression elevated photosynthetic efficiency during the early growth phase and triggered the expression of lipogenic genes. LPAT1 shows prokaryotic activity, which prefers the transfer of the 16-carbon acyl group to the sn-2 position on TAG. These results suggest that the plastidial TAG pathway is involved in enhancing cellular lipid content. Collectively, the results provide valuable insights that may be used to genetically improve lipid production by manipulating suitable metabolic targets.

## Additional file

**Additional file 1: Figure S1.** Protein structure of GPAT1 and LPAT1 obtained using SMART (http://smart.embl-heidelberg.de) and ChloroP (http://www.cbs.dtu.dk/services/ChloroP/). TM, transmembrane; SP, signal peptide; LPLAT and PlsC represented the conserved domains in proteins. **Figure S2.** Schematic representation of the expression vectors employed in this study. GPAT1 and LPAT1 genes were cloned into the expression vectors pHY18 (A) and pHY21 (C), respectively under the control of promoter PfcpC. An omega leader sequence and “ACC” nucleotides were inserted between the promoter and the target gene for boosting protein translation. For subcellular localization, GPAT1-EGFP (B) and LPAT1-EGFP (D) were employed under the control of promoter ProPtAP. **Figure S3.** Subcellular localization of GPAT1 and LPAT1 in *P. tricornutum* cells. A, Microscopy images of a representative wild-type cell; B, Microscopy images of a representative transgenic line with co-overexpression of GPAT1 and EGFP; C, Microscopy images of a representative transgenic line with co-overexpression of LPAT1 and EGFP. From left to right, fluorescence of EGFP, autofluorescence of chloroplasts, differential interference contrast (DIC), fluorescence images overlaid on DIC image. Scale bars represent 5 μm. **Figure S4.** PCR validation by agarose gel electrophoresis to verify the antibiotic gene *Shble* (349 bp), *CAT* (500 bp) and endogenous gene 18s rDNA (498 bp) PCR product. V1, pHY 21 vector (containing *Shble* gene); V2, pHY 18 vector (containing *CAT* gene); OE1 and OE2, individually overexpressed transformants; WT, wild type; B, negative control; M, marker. **Figure S5.** Glycolipid content in overexpression lines (OE1 and OE2) and wild-type (WT) harvested at day 4 and 7. A significant difference between WT and OE lines is indicated at the *p* < 0.05 (*) or *p* < 0.01 (**) level. Each value represents the mean ± SD (n = 3). **Figure S6.** Confocal microscopy images for detecting lipid droplet morphology in cells harvested on day 7. A, wild-type cells; B and C, transgenic lines. Left to right, fluorescence of Nile-red stained lipid droplets; autofluorescence of chloroplasts; fluorescence overlay; differential interference contrast (DIC). Scale bars represent 5 μm. **Figure S7.** Responses of wild-type (WT) and transgenic lines to sesamol treatment. Cells were harvested on day 7 and subjected to sesamol treatment for 48 h. A, Relative NADPH content in WT cells treated with different concentration of sesamol; B, Relative NADPH content in transgenic lines and WT treated with 1.5 mM sesamol; C, Relative neutral lipid content in transgenic lines and WT treated with 1.5 mM sesamol; D, *ME* expression level of transgenic lines and WT treated with 1.5 mM sesamol; E, ME enzyme activity of transgenic lines and WT treated with 1.5 mM sesamol. A significant difference between WT and OE lines is indicated at the *p* < 0.05 (*) or *p* < 0.01 (**) level. Each value represents the mean ± SD (n = 3). **Figure S8.** Phylogenetic tree showing relationship among LPATs from various organisms including plants (*A. thaliana*, *B. napus*, *R. communis*, *O. sativa*), microalgae (*P. tricornutum*, *C. reinhardtii*) and microbes (*E. coli*, *S. cerevisiae*). All the sequences were retrieved from NCBI and TAIR. The phylogenetic tree was established by MEGA7 using Maximum Likelihood method based on Poisson correction model. The percentages of replicate trees in which the associated taxa clustered together in the bootstrap test (1000 replicates) are represented along the branches. Red frame indicates ER-located CrLPAAT2 from *C. reinhardtii*; blue frame indicates plastidial LPAT1 from *P. tricornutum*. **Table S1.** Primers used in this study. **Table S2.** Subcellular localization of GPAT1 and LPAT1 predicted using HECTAR.

## Abbreviations

GPAT: glycerol-3-phosphate acyltransferase
LPAT: lysophosphatidic acid acyltransferase
AtpC: ATPase gamma subunit
PsbO: oxygen-evolving enhancer protein
PsbM: photosystem II subunit
PetJ: cytochrome c6
PAP: phosphatidic acid phosphatase
DGAT: diacylglycerol acyltransferase
ME: malic enzyme
G6PD: glucose-6-phosphate dehydrogenase
MGD: monogalactosyldiacylglycerol synthase
ACT: β-actin
