# Supplementary material for: Dual expression of plastidial GPAT1 and LPAT1 regulates triacylglycerol production and the fatty acid profile in Phaeodactylum tricornutum
Source: Biotechnol Biofuels. 2018 Nov 22;11:318. doi: 10.1186/s13068-018-1317-3 (PMC6249879; doi:10.1186/s13068-018-1317-3)
Supplement: Supplementary file 1 — Additional file 1: Figure S1. Protein structure of GPAT1 and LPAT1 obtained using SMART (http://smart.embl-heidelberg.de) and ChloroP (http://www.cbs.dtu.dk/services/ChloroP/). TM, transmembrane; SP, signal peptide; LPLAT and PlsC represented the conserved domains in proteins. Figure S2. Schematic representation of the expression vectors employed in this study. GPAT1 and LPAT1 genes were cloned into the expression vectors pHY18 (A) and pHY21 (C), respectively under the control of promoter PfcpC. An omega leader sequence and “ACC” nucleotides were inserted between the promoter and the target gene for boosting protein translation. For subcellular localization, GPAT1-EGFP (B) and LPAT1-EGFP (D) were employed under the control of promoter ProPtAP. Figure S3. Subcellular localization of GPAT1 and LPAT1 in P. tricornutum cells. A, Microscopy images of a representative wild-type cell; B, Microscopy images of a representative transgenic line with co-overexpression of GPAT1 and EGFP; C, Microscopy images of a representative transgenic line with co-overexpression of LPAT1 and EGFP. From left to right, fluorescence of EGFP, autofluorescence of chloroplasts, differential interference contrast (DIC), fluorescence images overlaid on DIC image. Scale bars represent 5 μm. Figure S4. PCR validation by agarose gel electrophoresis to verify the antibiotic gene Shble (349 bp), CAT (500 bp) and endogenous gene 18s rDNA (498 bp) PCR product. V1, pHY 21 vector (containing Shble gene); V2, pHY 18 vector (containing CAT gene); OE1 and OE2, individually overexpressed transformants; WT, wild type; B, negative control; M, marker. Figure S5. Glycolipid content in overexpression lines (OE1 and OE2) and wild-type (WT) harvested at day 4 and 7. A significant difference between WT and OE lines is indicated at the p < 0.05 (*) or p < 0.01 (**) level. Each value represents the mean ± SD (n = 3). Figure S6. Confocal microscopy images for detecting lipid droplet morphology in cells harvested on day 7. [file 13068_2018_1317_MOESM1_ESM.doc]

**
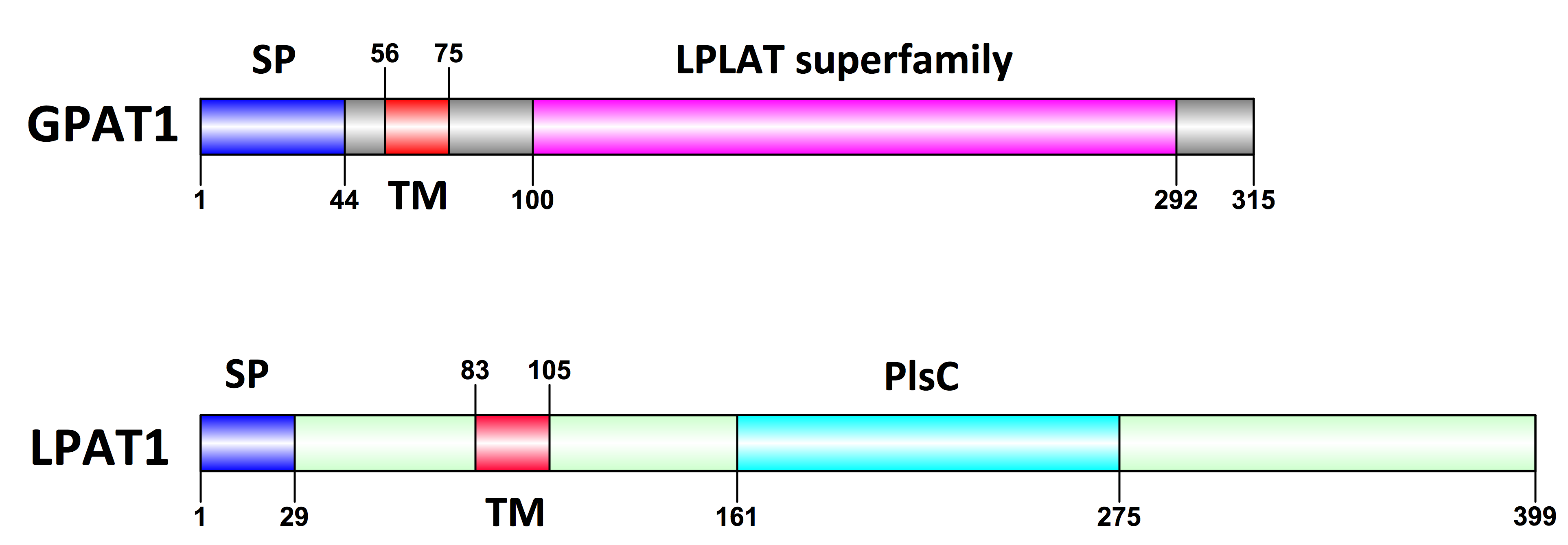
**

**Figure S1** Protein structure of GPAT1 and LPAT1 obtained by using SMART (http://smart.embl-heidelberg.de) and ChloroP (http://www.cbs.dtu.dk/services/ChloroP/). TM, transmembrane; SP, signal peptide; LPLAT and PlsC represented the conserved domains in proteins.


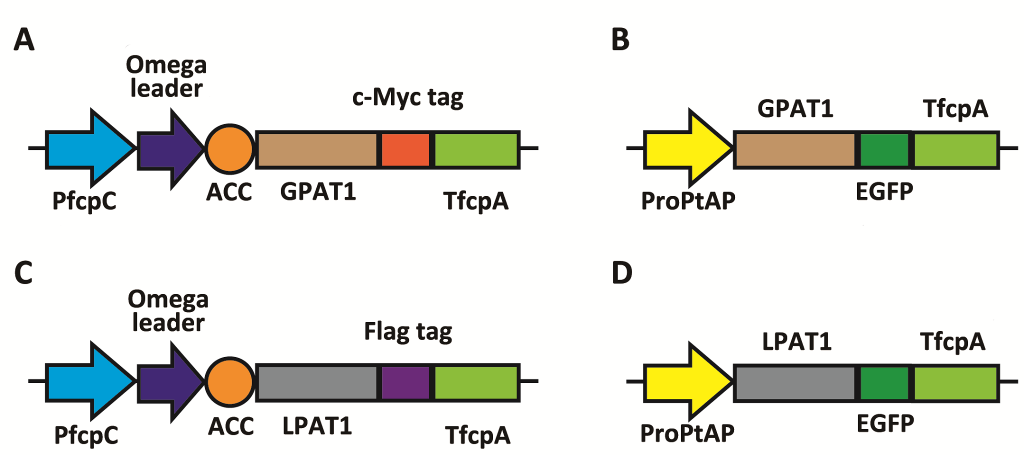


**Figure S2** Schematic representation of the expression vectors employed in this study. GPAT1 and LPAT1 genes were cloned into the expression vectors pHY18 (A) and pHY21 (C), respectively under the control of promoter PfcpC. An omega leader sequence and “ACC” nucleotides were inserted between the promoter and the target gene for boosting protein translation. For subcellular localization, GPAT1-EGFP (B) and LPAT1-EGFP (D) were employed under the control of promoter ProPtAP.


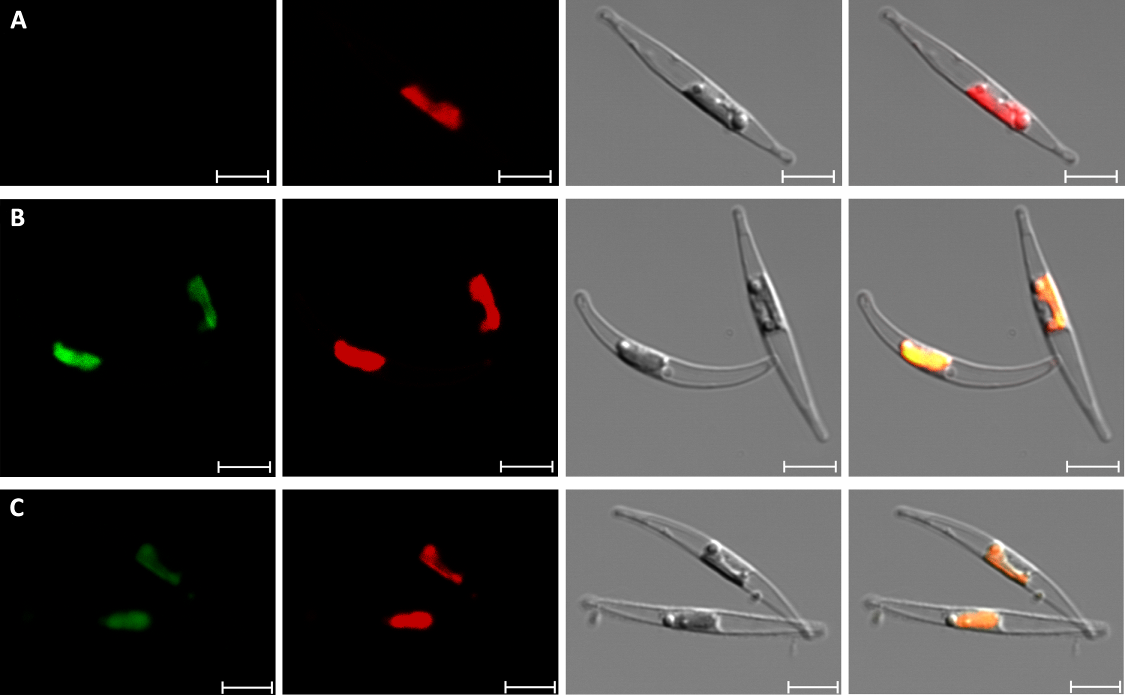


**Figure S3** Subcellular localization of GPAT1 and LPAT1 in *P. tricornutum* cells. A, Microscopy images of a representative wild-type cell; B, Microscopy images of a representative transgenic line with co-overexpression of GPAT1 and EGFP; C, Microscopy images of a representative transgenic line with co-overexpression of LPAT1 and EGFP. From left to right, fluorescence of EGFP, autofluorescence of chloroplasts, differential interference contrast (DIC), fluorescence images overlaid on DIC image. Scale bars represent 5 μm.


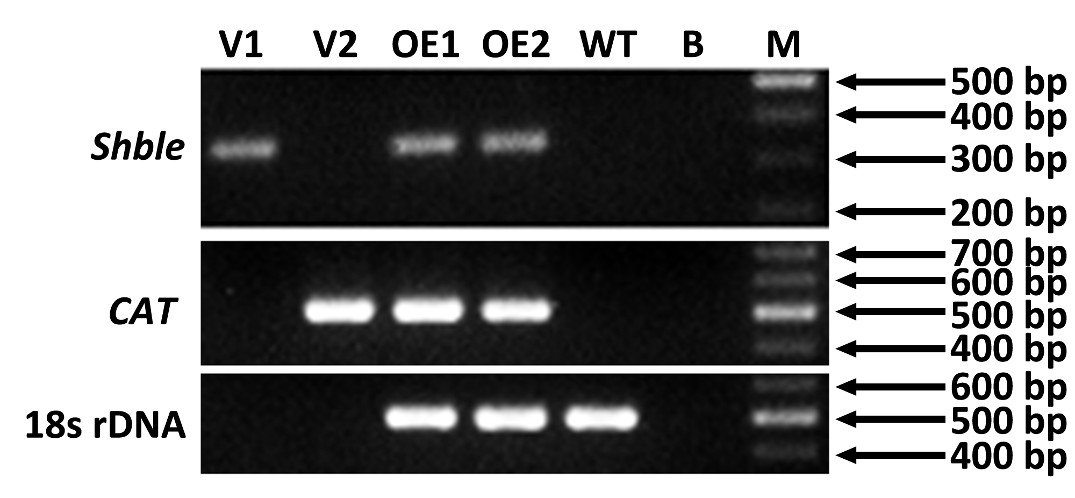


**Figure S4** PCR validation by agarose gel electrophoresis to verify the antibiotic gene *Shble* (349 bp), *CAT* (500 bp) and endogenous gene 18s rDNA (498 bp) PCR product. V1, pHY 21 vector (containing *Shble* gene); V2, pHY 18 vector (containing *CAT* gene); OE1 and OE2, individually overexpressed transformants; WT, wild type; B, negative control; M, marker.


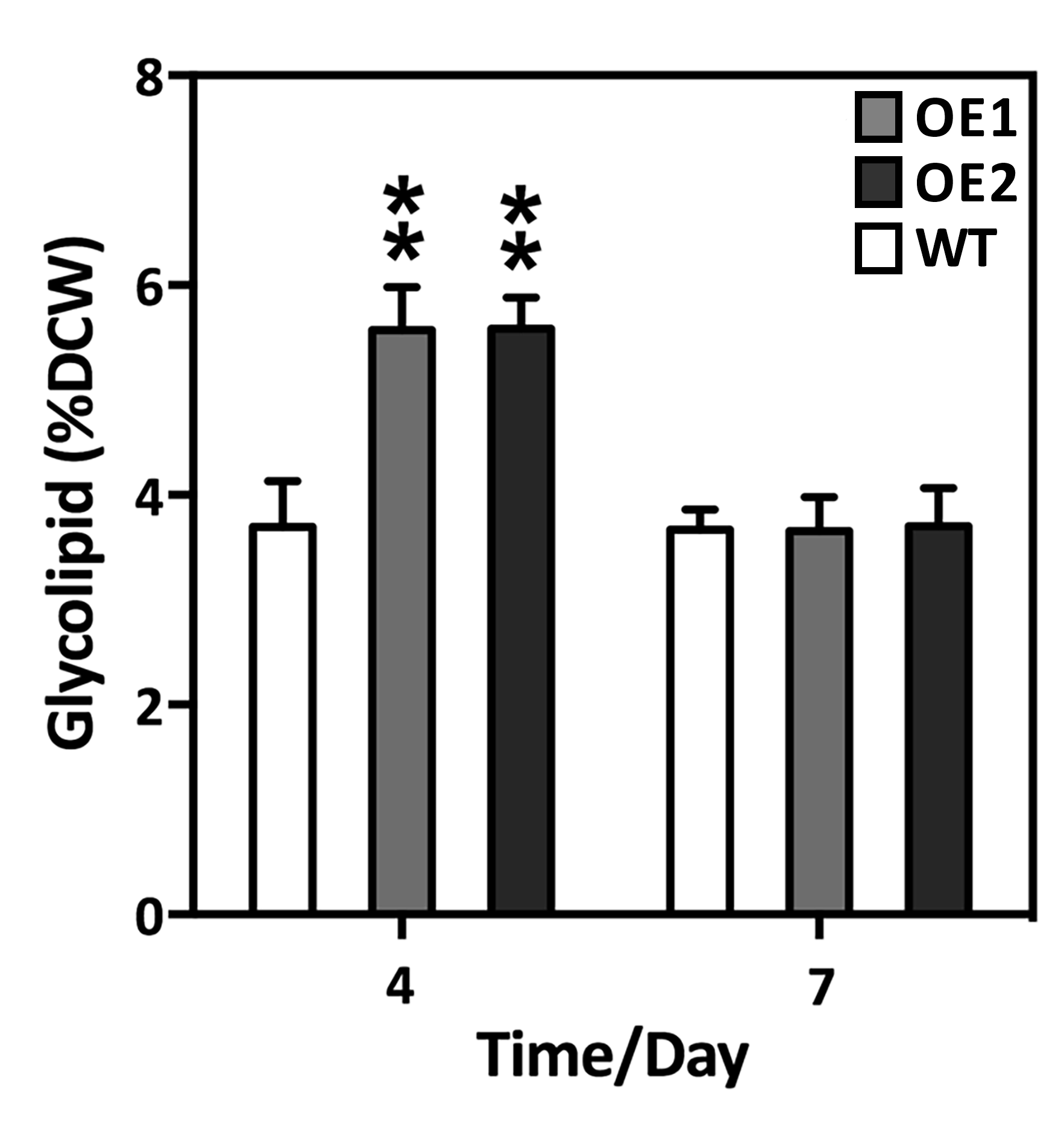


**Figure S5** Glycolipid content in overexpression lines (OE1 and OE2) and wild type (WT) harvested at day 4 and 7. A significant difference between WT and OE lines is indicated at the p < 0.05 (*) or p < 0.01 (**) level. Each value represents the mean ± SD (n = 3).


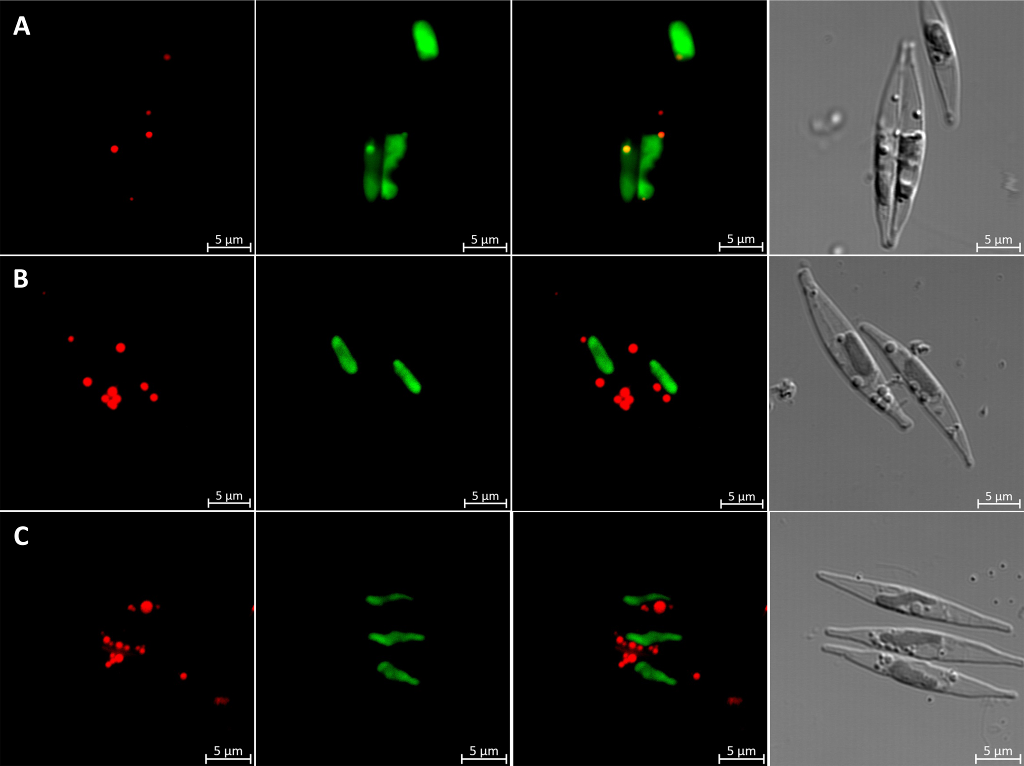


**Figure S6** Confocal microscopy images for detecting lipid droplet morphology in cells harvested on day 7. A, wild-type cells; B and C, transgenic lines. Left to right, fluorescence of Nile-red stained lipid droplets; autofluorescence of chloroplasts; fluorescence overlay; differential interference contrast (DIC). Scale bars represent 5 μm.


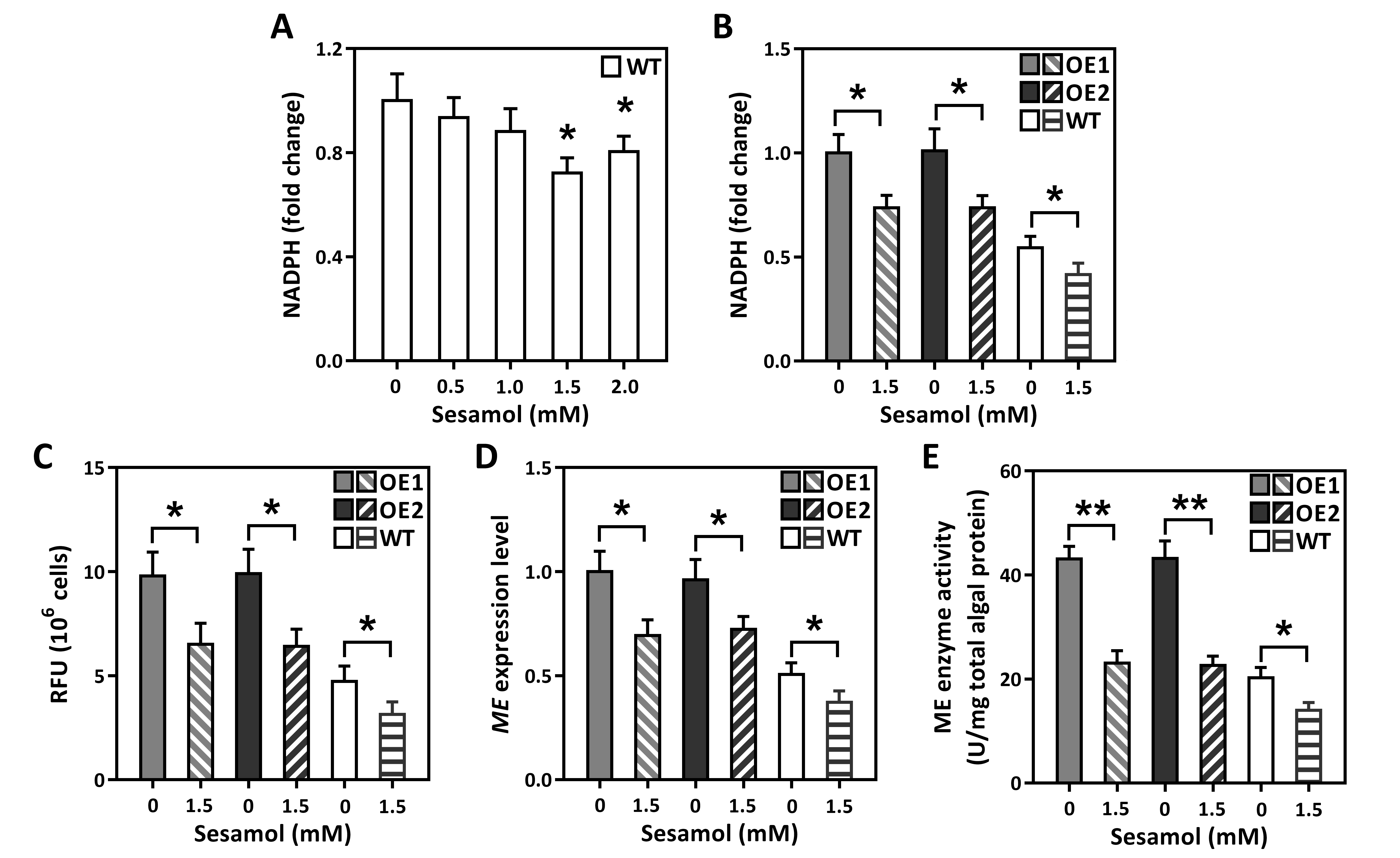


**Figure S7** Responses of wild-type (WT) and transgenic lines to sesamol treatment. Cells were harvested on day 7 and subjected to sesamol treatment for 48 h. A, Relative NADPH content in WT cells treated with different concentration of sesamol; B, Relative NADPH content in transgenic lines and WT treated with 1.5 mM sesamol; C, Relative neutral lipid content in transgenic lines and WT treated with 1.5 mM sesamol; D, *ME* expression level of transgenic lines and WT treated with 1.5 mM sesamol; E, ME enzyme activity of transgenic lines and WT treated with 1.5 mM sesamol. A significant difference between WT and OE lines is indicated at the *p* < 0.05 (*) or *p* < 0.01 (**) level. Each value represents the mean ± SD (n = 3).


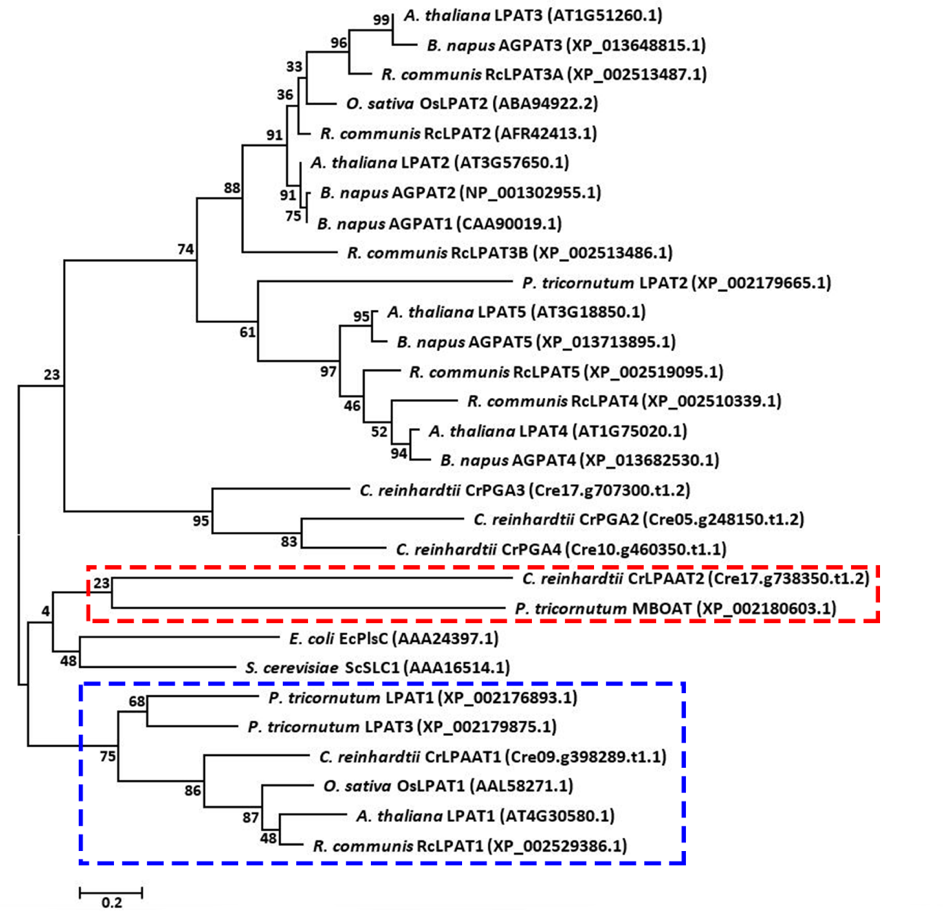


**Figure S8** Phylogenetic tree showing relationship among LPATs from various organisms including plants (*A. thaliana*, *B. napus*, *R. communis*, *O. sativa*), microalgae (*P. tricornutum*, *C. reinhardtii*) and microbes (*E. coli*, *S. cerevisiae*). All the sequences were retrieved from NCBI and TAIR. The phylogenetic tree was established by MEGA7 using Maximum Likelihood method based on Poisson correction model. The percentages of replicate trees in which the associated taxa clustered together in the bootstrap test (1000 replicates) are represented along the branches. Red frame indicates ER-located CrLPAAT2 from *C. reinhardtii*; blue frame indicates plastidial LPAT1 from *P. tricornutum*.

**Table S1** Primers used in this study.

| Gene name | Accession No. | Primer name | Sequence (5’→3’) |
| --- | --- | --- | --- |
| GPAT1 | XP_002177014.1 | GPAT1-f | ACAATTACAATCCAGTGGTACCATGGACTTATCTACGGTTCAATCAG |
|  |  | GPAT1-r | GAGTTTTTGTTCCAGGTGTTCTTGTCGGAGTCGTTG |
|  |  | q-GPAT1-f | ACGACAAGGTCGGAACAAAC |
|  |  | q-GPAT1-r | TAAAGGCACCGTCCTTGAAC |
| LPAT1 | XP_002176893.1 | LPAT1-f | ACAATTACAATCCAGTGGTACCATGAGGCATTTGAGAGGCGTAC |
|  |  | LPAT1-r | GTCCTTGTAGTCCAGGTGTGGTACAGTAGTCTCCTCCGT |
|  |  | q-LPAT1-f | TACCGATATGATGGAGATGG |
|  |  | q-LPAT1-r | AGACTACCTTATTACCTTGGG |
| AtpC | XP_002180505.1 | q-AtpC-f | CAGTATTCGTACCTTGGT |
|  |  | q-AtpC-r | AGAAGTGAGTTGGAAGAC |
| PsbO | XP_002180309.1 | q-PsbO-f | TGTCAATTCCAAGGTCAT |
|  |  | q-PsbO-r | TAGTGGCAGCATAATCAA |
| PsbM | XP_002184128.1 | q-PsbM-f | GTCCAATTCGGTGCCTAC |
|  |  | q-PsbM-r | TGGATGAATAAGTTGATGAGGAA |
| PetJ | XP_002186138.1 | q-PetJ-f | CATTATCTCCCAGGTCACC |
|  |  | q-PetJ-r | CAACATTGGCAATTTCTTCG |
| GPAT2 | XP_002181654.1 | q-GPAT2-f | AAGACTACCAAGTGAATG |
|  |  | q-GPAT2-r | TGAACTATATGATACGGAAG |
| GPAT3 | XP_002184838.1 | q-GPAT3-f | GCACCCTTTGACTCTAAA |
|  |  | q-GPAT3-r | GAGATCGTAACTGACCAT |
| LPAT2 | XP_002179665.1 | q-LPAT2-f | CTTCCACCTTCTATTGAG |
|  |  | q-LPAT2-r | TTGATTCGGATGTGTATT |
| LPAT3 | XP_002179875.1 | q-LPAT3-f | CAATTCCTGGATGGATATAC |
|  |  | q-LPAT3-r | GGACCTTACTGAGTTCTT |
| PAP | XP_002183995.1 | q-PAP-f | CCATCTTGTTCGGATTATTCG |
|  |  | q-PAP-r | CGTTGTGTTGGATCGTTG |
| DGAT1 | XP_002177753.1 | q-DGAT1-f | TGTGATTTCCGTTTCCATGACT |
|  |  | q-DGAT1-r | AAGAGGTTCAGATACAAATGGAAATATG |
| DGAT2A | XP_002184226.1 | q-DGAT2A-f | GATCTGGCCTAAATCCGTCA |
|  |  | q-DGAT2A-r | CGACGATGAGACGATCAAGA |
| DGAT2B | JQ837823.1 | q-DGAT2B-f | GACTCATCTCCCCGCTCAT |
|  |  | q-DGAT2B-r | AAACGACAGTGCTCCATGC |
| DGAT2D | XP_002177637.1 | q-DGAT2D-f | CCACTGTGCTGGGGAAGATA |
|  |  | q-DGAT2D-r | GCAGATGAGCCTTGTCAACC |
| ME | XP_002180331.1 | q-ME-f | TATGAATGGACCGATGGGCG |
|  |  | q-ME-r | TACATGCAACCGACGTCCAA |
| G6PD | XP_002183714.1 | q-G6PD-f | TGACCGCTACGGCATCATAC |
|  |  | q-G6PD-r | GCACATTCCTCCACGTCTCA |
| MGD | XP_002181685.1 | q-MGD-f | CAGAATACAGTGCGATATTG |
|  |  | q-MGD-r | TCGATTCAACCAGATACCAA |
| ACT | XP_002183424.1 | q-ACT-f | AGGCAAAGCGTGGTGTTCTTA |
|  |  | q-ACT-r | TCTGGGGAGCCTCAGTCAATA |
| Shble | - | Shble-f | CAAGTTGACCAGTGCCGTTC |
|  |  | Shble-r | GAAGTGCACGCAGTTGCC |
| AMP | - | AMP-f | TTCAACATTTCCGTGTCGCC |
|  |  | AMP-r | TTCATTCAGCTCCGGTTCCC |
| 18s rDNA | - | 18s-f | GCCATCCTTGGGTGGAATCA |
|  |  | 18s-r | ATTAAGCCGCAAGCTCCACT |

**Table S2** Subcellular localization of GPAT1 and LPAT1 predicted by using HECTAR.

| Protein Abbr. | Predicted targeting category | Signal peptide score | Signal peptide cleavage site | Type II signal anchor score | Chloroplast score | Mitochondrion score | Other score |
| --- | --- | --- | --- | --- | --- | --- | --- |
| GPAT1 | other localisation | 0.3030 | - | 0.2361 | - | 0.2491 | 0.7509 |
| LPAT1 | signal peptide | 0.7025 | 22 | 0.0577 | 0.0240 | - | - |
